# Panhypopituitarism due to Absence of the Pituitary Stalk: A Rare Aetiology of Liver Cirrhosis

**DOI:** 10.1155/2016/9071097

**Published:** 2016-04-24

**Authors:** Marta Gonzalez Rozas, Lidia Hernanz Roman, Diego Gonzalez Gonzalez, José Luis Pérez-Castrillón

**Affiliations:** ^1^Internal Department, Hospital de Segovia, Segovia, Spain; ^2^Internal Department, Hospital Universitario Río Hortega, Valladolid, Spain; ^3^Pathology Department, Hospital Universitario Río Hortega, Valladolid, Spain

## Abstract

Studies have established a relationship between hypothalamic-pituitary dysfunction and the onset of liver damage, which may occasionally progress to cirrhosis. Patients with hypopituitarism can develop a metabolic syndrome-like phenotype. Insulin resistance is the main pathophysiological axis of metabolic syndrome and is the causal factor in the development of nonalcoholic fatty liver disease (NAFLD). We present the case of a young patient with liver cirrhosis of unknown aetiology that was finally attributed to panhypopituitarism.

## 1. Introduction

Studies have established a relationship between hypothalamic-pituitary dysfunction and the onset of liver damage, which may occasionally progress to cirrhosis. Patients with hypopituitarism develop a metabolic syndrome-like phenotype, including secondary hormonal alterations, central obesity, insulin resistance, diabetes mellitus, dyslipidaemia, and, occasionally, hyperphagia [1, 2]. Insulin resistance is the main pathophysiological axis of metabolic syndrome and is the causal factor in the development of nonalcoholic fatty liver disease (NAFLD), which may evolve independently from liver cirrhosis.

We present the case of a young patient with liver cirrhosis of unknown aetiology that was finally attributed to panhypopituitarism.

## 2. Case Study

A 24-year-old man attended our hospital with fever of two days of evolution accompanied by chills and periumbilical abdominal pain, with no other associated clinical features, except for occasional episodes of epistaxis and gingival bleeding. The medical history was remarkable only for hepatitis in childhood.

At admission, the patient was conscious and oriented. Physical examination revealed obesity (body mass index [BMI] = 30), height 174 cm, waist circumference 117 cm, blood pressure systolic 127 mmHg, blood pressure diastolic 75 mmHg, cutaneous-mucous paleness, hepatomegaly (3 cm), hypermobility in the lower limbs, and macrodactylia, without other remarkable features.

Laboratory tests showed leukocytes 7.2 × 1000/*μ*L, haemoglobin 12.9 g/dL, mean corpuscular volume 88.5 fl, and platelets 98 × 1000/*μ*L. Clotting and blood smears were normal. Biochemical tests showed glucose 97 mg/dL, HbA1c 4.3%, urea 43 mg/dL, total cholesterol 201 mg/dL, triglycerides 125 mg/dL, uric acid 7.91 mg/dL, creatinine 1.6 mg/dL, total bilirubin 1.29 mg/dL, calcium 9.9 mg/dL, glutamic-oxaloacetic transaminase (GOT) 30 U/L, glutamic-pyruvic transaminase (GPT) 58 U/L, alkaline phosphatase 139 U/L, sodium 141 mEq/L, and potassium 4 mEq/L.

Serology for hepatitis B and C, cytomegalovirus, and toxoplasma was negative. Autoimmune tests were negative for antinuclear antibodies, anti-smooth muscle antibodies, and antimitochondrial antibodies. Other causes of chronic liver disease such as drug-induced and cholestatic liver disease and metabolic disease were ruled out (alpha-antitrypsin (199 mg/dL), ceruloplasmin (38 mg/dL), and copper (127 mg/dL) were normal).

Hormone testing showed ACTH 14.5 pg/mL, GH < 0.11 ng/mL, somatomedin C 3.56 ng/mL, TSH 0 mUI/L, FT4 0.6 ng/dL, cortisol 1.4 *μ*g/dL, FSH 0.2 U/L, prolactin < 0.6 ng/mL, testosterone < 0.1 ng/dL, and insulin 19.8 *μ*UI/mL. A TRH and LH-FH test was performed with no response. The patient had a normal karyotype (46XY).

Chest X-ray, abdominal CT scan, electrocardiogram, and echocardiogram were normal. Abdominal ultrasound confirmed dilation of the portal vein (14 mm) and hepatosplenomegaly. Hip radiography showed bilateral hip dysplasia without closing of growth plates, and cerebral MRI showed absence of the pituitary stalk.

**Figure 1:**
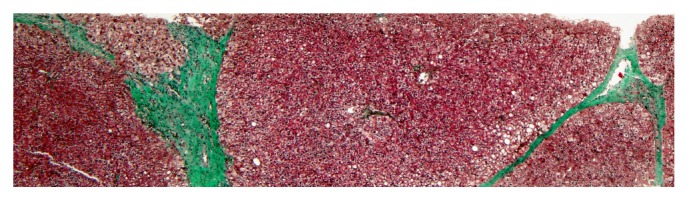
Liver biopsy. Architectural distortion with nodular areas bounded by fibrous tract (Masson).

Finally, liver biopsy showed architectural distortion with nodular areas bounded by fibrous tracts, with ductal proliferation without iron deposits, suggestive of liver cirrhosis with mild steatosis and minimal inflammatory activity (Figure 1).

The patient received hormone replacement therapy with cortisol, thyroid hormones, and testosterone.

## 3. Relationship between Cirrhosis and Panhypopituitarism

The first cases establishing an association between hypothalamic-pituitary dysfunction and liver damage were reported in 2004. Most cases occur in children or adolescents who present hypothalamic dysfunction secondary to structural lesions such as perinatal asphyxia and craniopharyngiomas or genetic disorders such as Prader-Willi disease [1–3].

NAFLD affects 20–50% of adults in developed countries and includes histological alterations that range from simple steatosis to nonalcoholic steatohepatitis (NASH) and cirrhosis. Simple steatosis is often associated with obesity and is characterized by fat accumulation in the liver, without inflammation, and is considered benign [4].

NASH occurs in 2-3% of cases and is characterized by steatosis, inflammation, and pericellular fibrosis that may progress to cirrhosis and hepatocellular carcinoma. The definitive diagnosis of NAFLD requires a liver biopsy [4]. NAFLD is characterized by insulin resistance, central obesity, and impaired glucose tolerance and is considered a hepatic manifestation of metabolic syndrome. The increased prevalence of obesity and diabetes has increased the incidence of NAFLD, which is now the leading cause of chronic liver disease in North America [5].

Furthermore, patients with hypopituitarism that have a growth hormone deficiency (GHD) and other hypothalamic dysfunctions show a similar phenotype. Due to the similarity of the two phenotypes, it is hypothesized that patients with hypothalamic-pituitary dysfunction might develop liver disease.

## 4. Pathogenesis of NAFLD

The pathogenesis of NAFLD is very complex and involves different mechanisms that suggest an association between adipose tissue and the liver. Three possible underlying mechanisms have been proposed: first, the excessive accumulation of lipids; second, an inflammatory response that causes cell apoptosis; and, third, a probable defect in the reparative response to the damage suffered. Hormonal deficiencies may contribute to the occurrence of any of these mechanisms.

Altered lipid homeostasis in the liver is a key point in the pathogenesis of NAFLD. Initially, it was thought that elevated levels of free fatty acids (FFA) were the main cause of cell damage, due to their ability to induce apoptosis, which promotes hepatocyte death [6].

Circulating FFA constitute 60% of body fat and correlate with the severity of NAFLD. A lipidomic analysis by Puri et al. showed that despite the increased hepatic lipid content, FFA levels were not altered and found high concentrations of triacylglycerol and diacylglycerol with an increase in saturated FFA, which are more hepatotoxic [7]. Other lipid abnormalities such as increased free cholesterol and reduced phosphatidylcholine are also involved [7].

The oxidation of FFA within hepatocytes is the main source of reactive oxygen species (ROS). When ROS production exceeds the antioxidant capacity of the cell this causes mitochondrial and nuclear DNA damage, disruption of the phospholipid membrane and the release of proinflammatory cytokines and toxic products that perpetuate the damage, causing cell death. Some of these products activate fibrogenic hepatic stellate cells and this continues the inflammatory process [8].

Different cytokines and adipokines, in addition to genetic factors, participate in the pathogenesis of NAFLD. Tumour necrosis factor (TNF-*α*) is a proinflammatory cytokine that is induced, in part by FFA, and, experimentally, seems to promote hepatic lipotoxicity [9]. Patients with NASH have higher levels of TNF-*α* than those with isolated steatosis, possibly caused by increased intestinal permeability that allows a high level of endotoxins in the systemic and portal circulation [10]. The imbalance in the inflammatory pathway mediated by TNF-*α* is important in the transition from NASH to hepatocellular carcinoma.

Adiponectin, an adipokine with anti-inflammatory, insulin-sensitizing, and antifibrotic properties, is reduced in patients with NASH and those with visceral obesity and insulin resistance. It exercises a hepatoprotective effect through inactivation of TNF-*α* synthesis.

Leptin, a hormone that regulates appetite and fat metabolism through the CNS, has a proinflammatory effect and stimulates adipocyte production of TNF-*α*. In animal models of fibrotic or fatty liver, it behaves as a profibrotic cytokine, while in humans with NAFLD it correlates with the severity of liver fibrosis, regardless of the degree of insulin resistance and the BMI [2, 11]. In patients with hypopituitarism and GHD, leptin levels are higher than those corresponding to their obesity [12].

## 5. Insulin Resistance and Hormone Deficiencies

Insulin resistance is closely linked to visceral obesity and metabolic syndrome and is clearly accepted as the central axis of the pathogenesis of NAFLD in the context of type 2 DM [13, 14]. The main metabolic changes that establish a relationship between hypopituitarism and cirrhosis are insulin resistance, the accumulation of hepatic triglycerides, and increased oxidative stress. In addition, GHD, insulin-like growth factor 1 (IGF-1) and other factors such as gonadotropins or cortisol are also involved [15].

In physiological conditions, insulin suppresses lipolysis and glucose production and promotes lipogenesis and the uptake, utilization, and storage of glucose [16]. Insulin resistance favours the mobilization and deposition of fatty acids outside the adipose tissue, reduces the inhibition of lipolysis, and increases* de novo* hepatic lipogenesis [17]. It increases the hepatic expression of fatty acid transport proteins and their reesterification [18] and produces alterations in the insulin receptor and the GLUT 4 transporter and in the phosphorylation of both substrates and insulin receptors. This increases oxidative stress, mitochondrial toxicity, and the dysregulation of adipokines with subsequent inflammation and, finally, fibrosis [19].

GH and insulin-like growth factor 1 (IGF-1) appear to play an important role in the regulation of hepatic lipid metabolism [20]. The mechanism by which their deficiency contributes to hepatic steatosis and fibrosis is not fully known. Reductions in or an absence of GH secretion in the anterior pituitary gland may cause a reduction in the hepatic secretion of IGF-1, which is secreted by the hepatocytes after stimulation by GH. IGF-1 is a catabolic hormone that plays an important role in protein synthesis and also stimulates IGFBP-3 secretion by the Kupffer cells. It has antifibrotic, cell-protective, insulin-like effects. Therefore, absence of or reduction in IGF-1 secretion would lead to increased hepatic glucose production and favour peripheral insulin resistance [21]. GH promotes lipolysis in adipose tissue and the secretion of very low density lipoproteins by the liver, and therefore low levels of GH promote severe hypertriglyceridemia in the liver [21].

Patients with GHD have greater fat infiltration than those without this deficiency and NAFLD patients have lower levels of GH, although this might reflect a decrease in GH due to obesity. Furthermore, it appears that the level of insulin resistance is higher in patients with GH deficiency than in healthy persons with the same BMI. Studies have found that NAFLD patients have lower serum GH [22] and IFG-1 [23].

Although GH is the major stimulant of IGF-1 synthesis in hepatocytes, other cytokines, such as interleukin 1 beta (IL-1*β*), TNF-*α*, and interleukin 6 (IL-6), inhibit IGF-1 secretion [21]. IGF-1 bioactivity is also reduced by high levels of IGFBP1-2, which act primarily by blocking the actions of IGF-1 [24].

The chronic liver disease was due, therefore, to IGF-1 deficiency and GH resistance, because the hepatic response to GH is diminished in the presence of liver disease [25]. Hepatic fibrosis is caused by activation of hepatic stellate cells which are activated by inflammatory cytokines.

Associations have been established between different hormones and NAFLD, although clinical data is scarce and less validated. There is an association between liver steatosis and low testosterone levels, probably due to increased BMI and waist circumference [26]. Testosterone therapy decreases the accumulation of liver fat measured by CT scan [27], while oestrogen appears to protect against the development of NAFLD [28]. Glucocorticoids appear to increase FFA levels and hypothyroidism [29], while low vitamin D levels [30] are associated with the development of NAFLD. Glucagon-like peptide (GLP-1) is secreted by L cells in the small intestine and reduces intrahepatic lipid accumulation via its incretin effect, increasing the secretion of insulin-dependent glucose and a reduction in pancreatic beta cells and the appetite [31].

In patients with hypothalamic and pituitary dysfunction, NAFLD develops relatively rapidly, with a high prevalence of cirrhosis, and is a serious complication in patients with GH, IGF-1, and IGFBP3 deficiencies.

## 6. Treatment

GH replacement therapy improves the hepatic process and dyslipidaemia and has a protective endothelial effect in patients with GH deficiency, although the effects are minor, probably due to the persistence of GH resistance. It also seems to have a direct or indirect effect on the reduction of hepatic oxidative stress. Furthermore, it reduces levels of C-reactive protein (CRP) and TNF-*α*, which play an important role in inflammation and insulin resistance [20, 21].

IGF1 overexpression or supplementation attenuates fibrogenesis in mouse models. It improves hepatocellular function, promotes liver regeneration, reduces oxidative damage increases albumin, and has protective effects on the endothelium and vascular cells. It also has extrahepatic effects such as increased food intake, muscle mass, bone density, and gonadal function. Treatment with recombinant human IGF-1, which seems to reverse fibrotic effects, is beginning to be used in patients with cirrhosis, although further studies on its use are required [24, 32].

Other treatments aimed at treating NASH are those used in the treatment of the components of the metabolic syndrome: hypertension, obesity, dyslipidaemia, and insulin resistance. Novel treatments such as caspase inhibition, agonism/antagonism of the adenosine system, PPAR alpha and delta, peripheral cannabinoid 1 receptor agonism, farnesoid x receptor agonism, monoclonal antibodies to TNF-*α*, thyroid hormones analogues, and enzymatic modulation are under investigation [33].

Our patient had a GH deficiency, among others, in the context of hypopituitarism with abdominal obesity. Insulin resistance and hormonal deficiencies contributed to the rapid development of cirrhosis, which improved with replacement therapy.

## References

[1] Nakajima K., Hashimoto E., Kaneda H. (2005). Pediatric nonalcoholic steatohepatitis associated with hypopituitarism. *Journal of Gastroenterology*.

[2] Adams L. A., Feldstein A., Lindor K. D., Angulo P. (2004). Nonalcoholic fatty liver disease among patients with hypothalamic and pituitary dysfunction. *Hepatology*.

[3] Nyunt A., Kochar N., Pilz D. T., Kingham J. G. C., Jones M. K. (2005). Adult cirrhosis due to untreated congenital hypopituitarism. *Journal of the Royal Society of Medicine*.

[4] Ahima R. S. (2008). The natural history of nonalcoholic fatty liver disease: insights from children and mice. *Gastroenterology*.

[5] Gholam P. M., Flancbaum L., Machan J. T., Charney D. A., Kotler D. P. (2007). Nonalcoholic fatty liver disease in severely obese subjects. *The American Journal of Gastroenterology*.

[6] Feldstein A. E., Canbay A., Guicciardi M. E., Higuchi H., Bronk S. F., Gores G. J. (2003). Diet associated hepatic steatosis sensitizes to Fas mediated liver injury in mice. *Journal of Hepatology*.

[7] Puri P., Baillie R. A., Wiest M. M. (2007). A lipidomic analysis of nonalcoholic fatty liver disease. *Hepatology*.

[8] Browning J. D., Horton J. D. (2004). Molecular mediators of hepatic steatosis and liver injury. *The Journal of Clinical Investigation*.

[9] Feldstein A. E., Werneburg N. W., Canbay A. (2004). Free fatty acids promote hepatic lipotoxicity by stimulating TNF-*α* expression via a lysosomal pathway. *Hepatology*.

[10] Crespo J., Cayón A., Fernández-Gil P. (2001). Gene expression of tumor necrosis factor *α* and TNF-receptors, p55 and p75, in nonalcoholic steatohepatitis patients. *Hepatology*.

[11] Ikejima K., Takei Y., Honda H. (2002). Leptin receptor-mediated signaling regulates hepatic fibrogenesis and remodeling of extracellular matrix in the rat. *Gastroenterology*.

[12] Al-Shoumer K. A. S., Anyaoku V., Richmond W., Johnston D. G. (1997). Elevated leptin concentrations in growth hormone-deficient hypopituitary adults. *Clinical Endocrinology*.

[13] Masuoka H. C., Chalasani N. (2013). Nonalcoholic fatty liver disease: an emerging threat to obese and diabetic individuals. *Annals of the New York Academy of Sciences*.

[14] Doycheva I., Patel N., Peterson M., Loomba R. (2013). Prognostic implication of liver histology in patients with nonalcoholic fatty liver disease in diabetes. *Journal of Diabetes and Its Complications*.

[15] Takahashi Y., Iida K., Takahashi K. (2007). Growth hormone reverses nonalcoholic steatohepatitis in a patient with adult growth hormone deficiency. *Gastroenterology*.

[16] Saltiel A. R., Kahn C. R. (2001). Insulin signalling and the regulation of glucose and lipid metabolism. *Nature*.

[17] Utzschneider K. M., Kahn S. E. (2006). The role of insulin resistance in nonalcoholic fatty liver disease. *The Journal of Clinical Endocrinology & Metabolism*.

[18] Miquilena-Colina M. E., Lima-Cabello E., Sánchez-Campos S. (2011). Hepatic fatty acid translocase CD36 upregulation is associated with insulin resistance, hyperinsulinaemia and increased steatosis in non-alcoholic steatohepatitis and chronic hepatitis C. *Gut*.

[19] Sanyal A. J., Campbell-Sargent C., Mirshahi F. (2001). Nonalcoholic steatohepatitis: association of insulin resistance and mitochondrial abnormalities. *Gastroenterology*.

[20] Takahashi Y. (2012). Essential roles of growth hormone (GH) and insulin-like growth factor-I (IGF-I) in the liver. *Endocrine Journal*.

[21] Ichikawa T., Nakao K., Hamasaki K. (2007). Role of growth hormone, insulin-like growth factor 1 and insulin-like growth factor-binding protein 3 in development of non-alcoholic fatty liver disease. *Hepatology International*.

[22] Xu L., Xu C., Yu C. (2012). Association between serum growth hormone levels and nonalcoholic fatty liver disease: a cross-sectional Study. *PLoS ONE*.

[23] Fusco A., Miele L., D'Uonnolo A. (2012). Nonalcoholic fatty liver disease is associated with increased GHBP and reduced GH/IGF-I levels. *Clinical Endocrinology*.

[24] Bonefeld K., Møller S. (2011). Insulin-like growth factor-I and the liver. *Liver International*.

[25] Lonardo A., Loria P., Leonardi F., Ganazzi D., Carulli N. (2002). Growth hormone plasma levels in nonalcoholic fatty liver disease. *American Journal of Gastroenterology*.

[26] Kim S., Kwon H., Park J.-H. (2012). A low level of serum total testosterone is independently associated with nonalcoholic fatty liver disease. *BMC Gastroenterology*.

[27] Hoyos C. M., Yee B. J., Phillips C. L., Machan E. A., Grunstein R. R., Liu P. Y. (2012). Body compositional and cardiometabolic effects of testosterone therapy in obese men with severe obstructive sleep apnoea: a randomised placebo-controlled trial. *European Journal of Endocrinology*.

[28] Tian G.-X., Sun Y., Pang C.-J. (2012). Oestradiol is a protective factor for non-alcoholic fatty liver disease in healthy men. *Obesity Reviews*.

[29] Ittermann T., Haring R., Wallaschofski H. (2012). Inverse association between serum free thyroxine levels and hepatic steatosis: results from the study of health in pomerania. *Thyroid*.

[30] Rhee E.-J., Kim M. K., Park S. E. (2013). High serum vitamin D levels reduce the risk for nonalcoholic fatty liver disease in healthy men independent of metabolic syndrome. *Endocrine Journal*.

[31] Mells J. E., Fu P. P., Sharma S. (2012). Glp-1 analog, liraglutide, ameliorates hepatic steatosis and cardiac hypertrophy in C57BL/6J mice fed a western diet. *American Journal of Physiology—Gastrointestinal and Liver Physiology*.

[32] Conchillo M., de Knegt R. J., Payeras M. (2005). Insulin-like growth factor I (IGF-I) replacement therapy increases albumin concentration in liver cirrhosis: results of a pilot randomized controlled clinical trial. *Journal of Hepatology*.

[33] Federico A., Zulli C., de Sio I. (2014). Focus on emerging drugs for the treatment of patients with non-alcoholic fatty liver disease. *World Journal of Gastroenterology*.

